# Supplementary material for: SAMPLE: An R Package to Estimate Sampling Effort for Species' Occurrence Rates
Source: Ecol Evol. 2025 Mar 27;15(4):e70998. doi: 10.1002/ece3.70998 (PMC11949539; doi:10.1002/ece3.70998)
Supplement: Supplementary file 3 — Data S2. Example data set used in this manuscript, which can also be used to verify the functionality of the package. [file ECE3-15-e70998-s003.pdf]

Supplementary Material

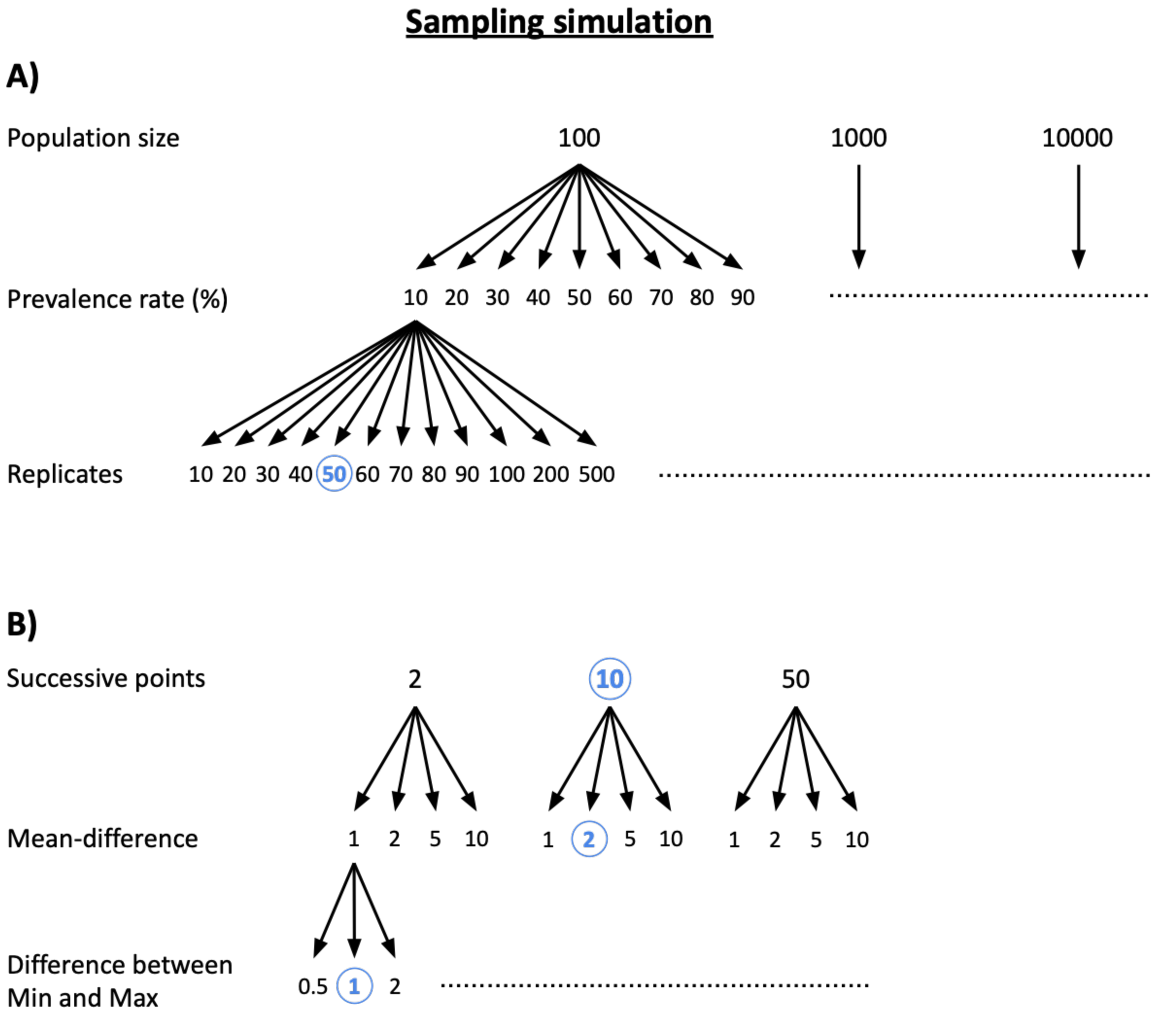

Fig. S1 Schematic representation of the simulation sampling, where: different sized populations were sampled with different prevalence rates and different number of replicates **(A)**; and one example of a population with 1000 individuals, a prevalence rate of 50% and 50 replicates was then taken and ran with different settings of successive points, mean-difference and Δ **(B).** A final example was chosen and ran 10 times with the exact same settings and using the default values highlighted in blue in order to evaluate the natural variation in the process.

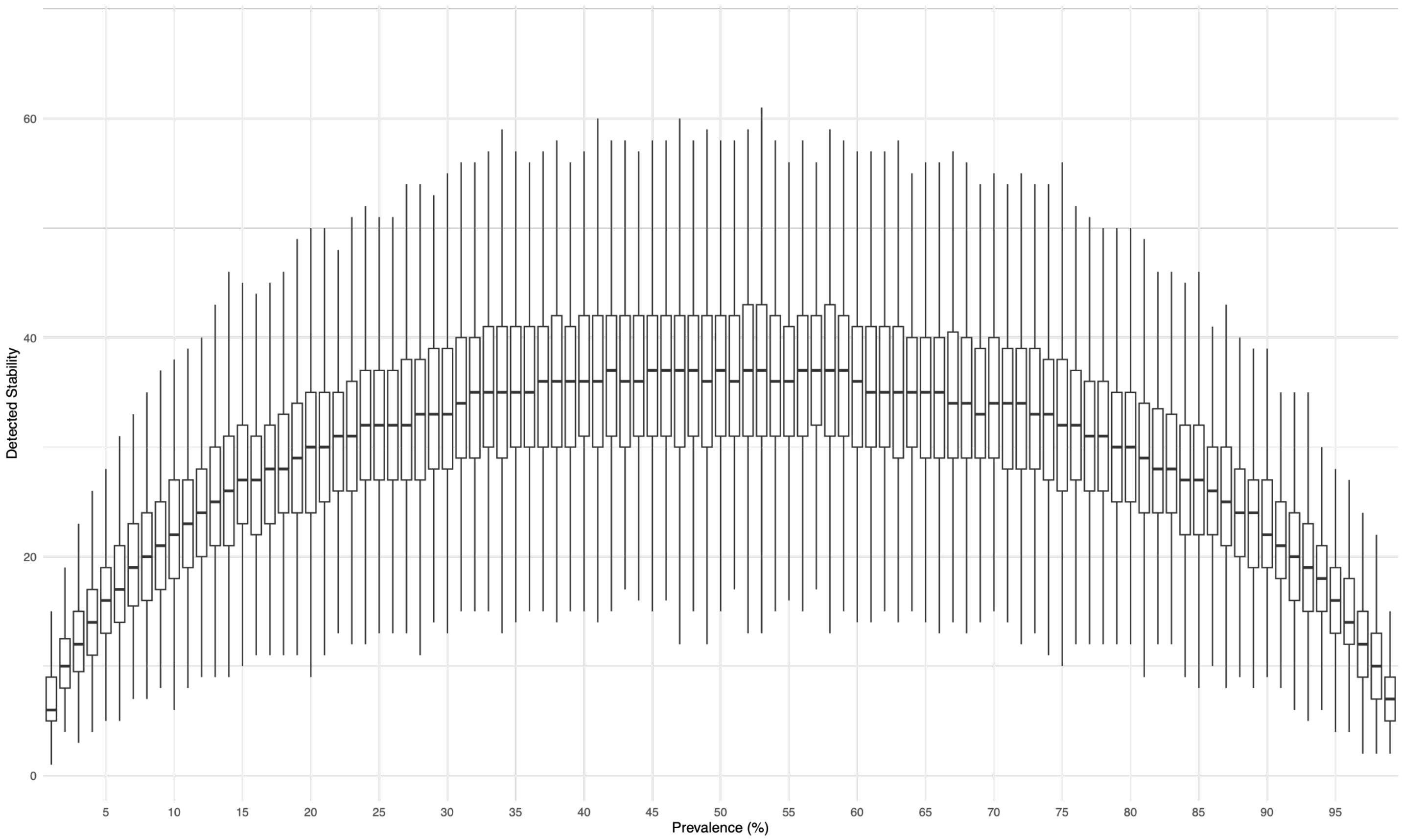

Fig. S2 Boxplots representing the number of individuals required to detect prevalence stability across different prevalence rates (1%–99%), based on samples of 100 individuals. Each boxplot corresponds to 1,000 replicate simulations, with the y-axis showing the sample size at which SAMPLE identified stability in the prevalence estimate.

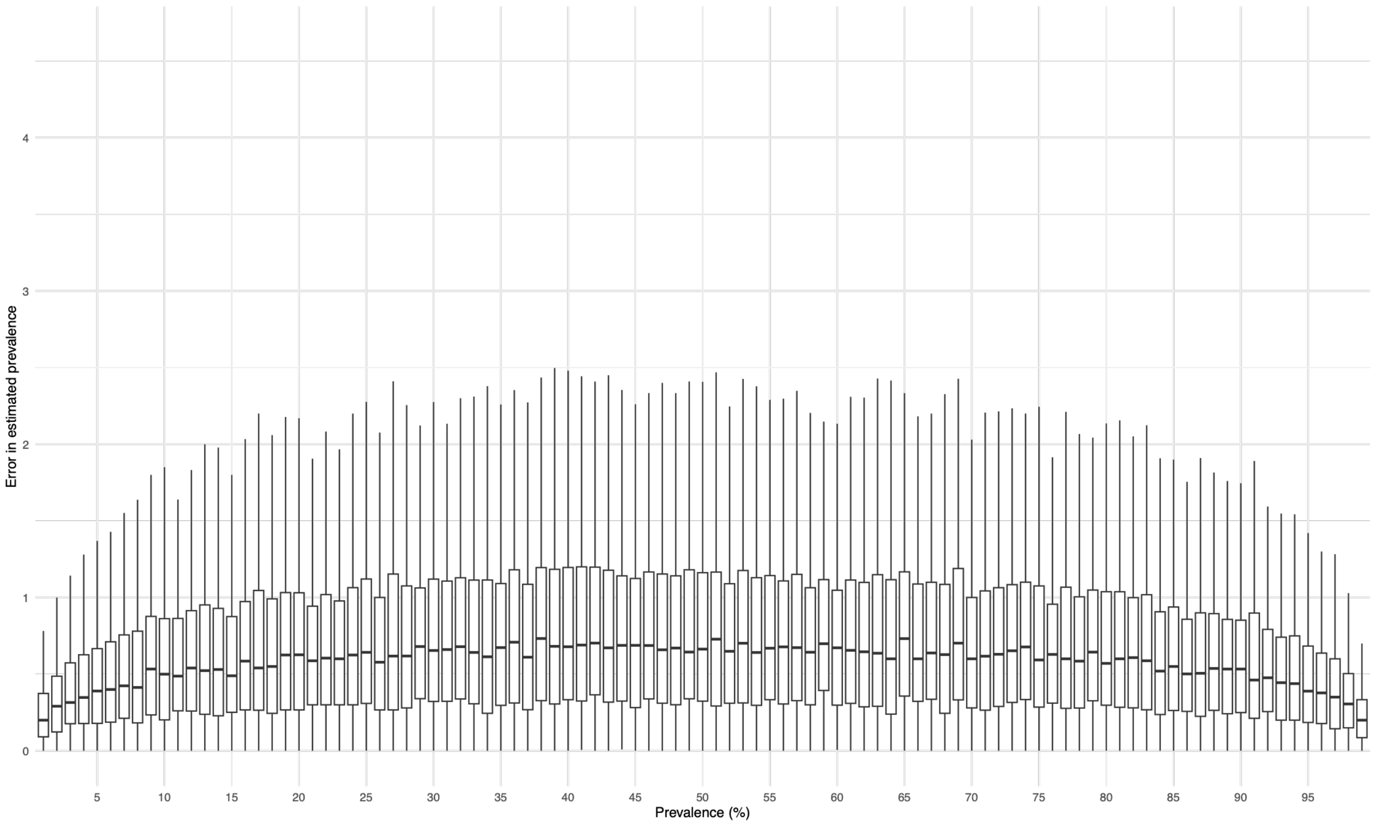

Fig. S3 Boxplots representing the error (%) between the prevalence estimated by SAMPLE and the true prevalence, based on samples of 100 individuals. The x-axis shows the true simulated prevalence, ranging from 1% to 99%. Each boxplot corresponds to 1,000 replicates.

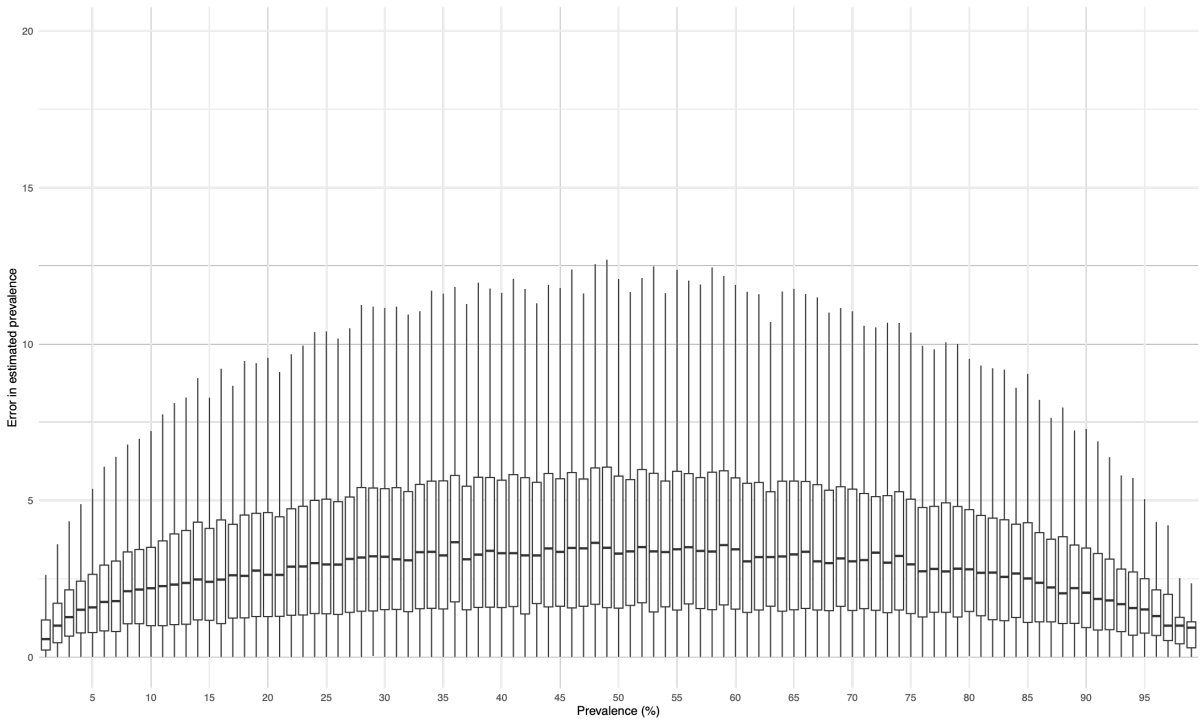

Fig. S4 Boxplots showing the error (%) between the prevalence estimated by SAMPLE and the true prevalence in a simulated population of 100,000 individuals. For each simulation, a random sample of 100 individuals (without replacement) was drawn, and SAMPLE was applied to estimate prevalence. The x-axis represents the true prevalence in the population, ranging from 1% to 99%. Each boxplot is based on 1,000 replicate simulations.

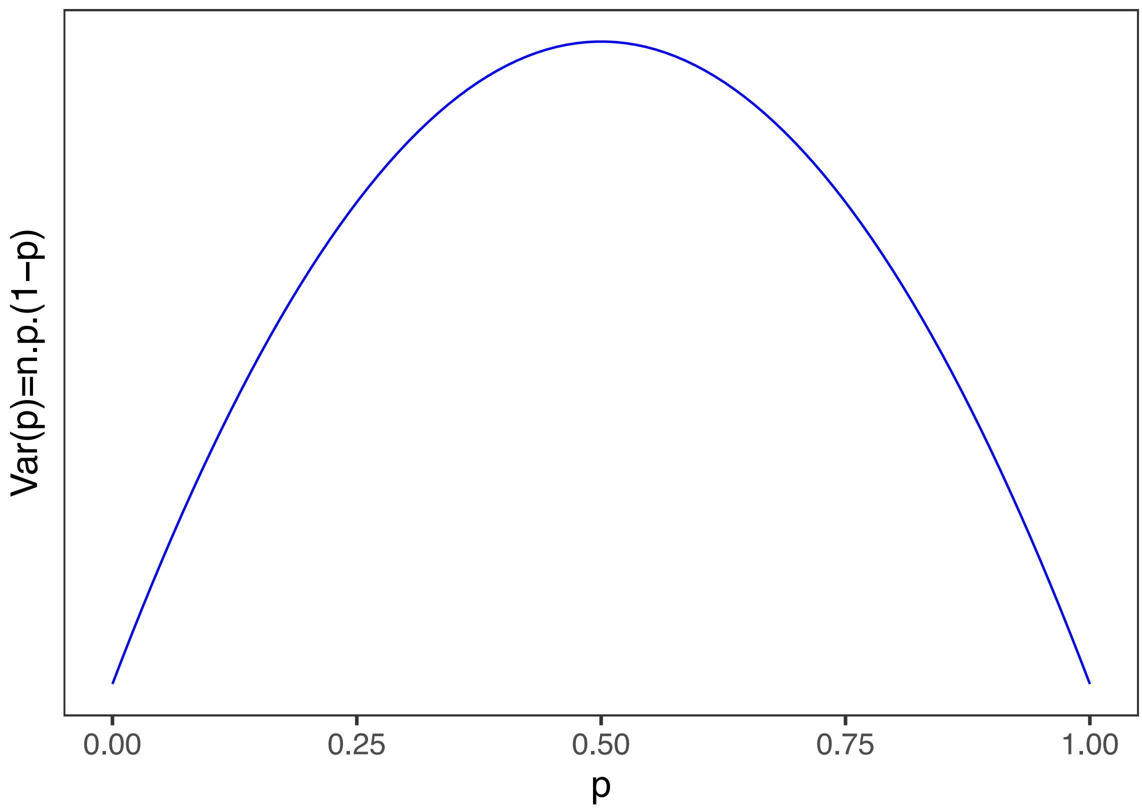

Fig. S5 Variance of a binomial distribution as a function of the true prevalence (p). The plot illustrates the relationship v(p) = n.p.(1-p), where n is the sample size. The U-shape demonstrates that variance is lowest when the p is near its extremes (0 or 1) and highest when p is 0.5, indicating maximum variability. Specific values are not shown on the y-axis because the variance depends on the sample size *n*, which is not fixed in this general representation.

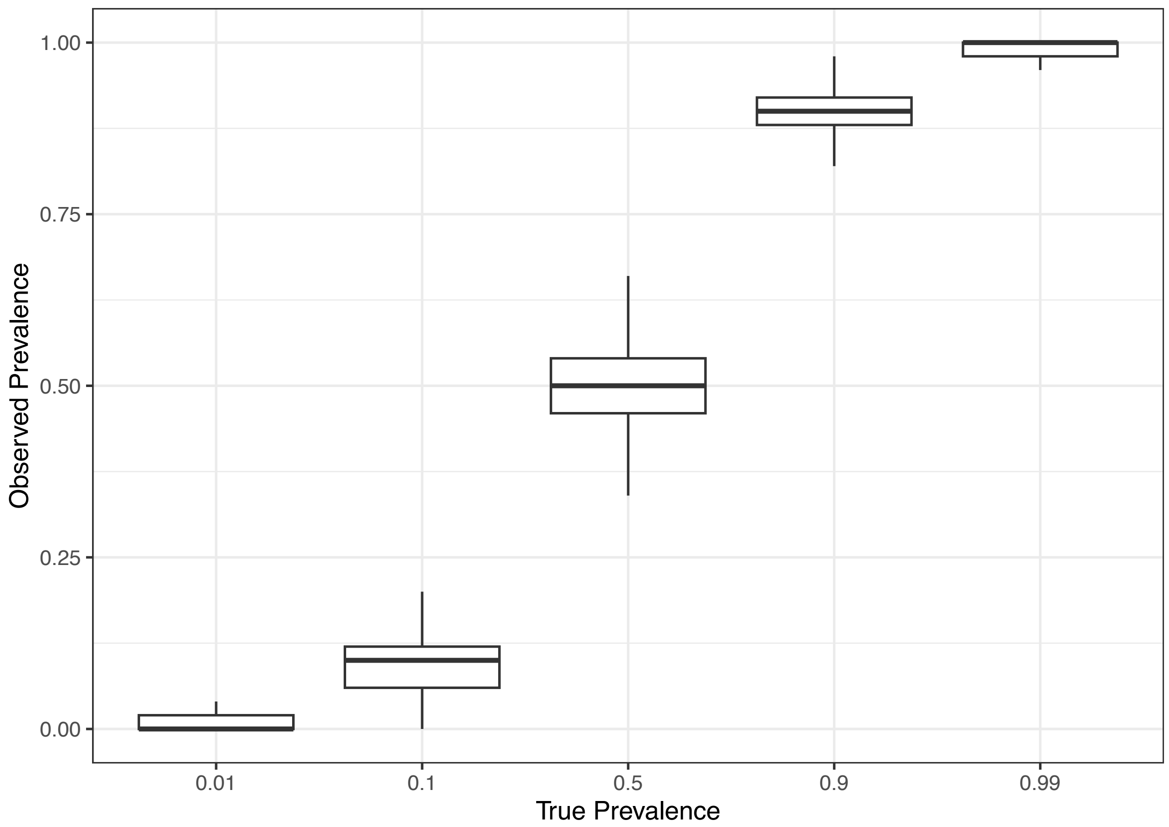

Fig. S6: Observed prevalence estimates derived from 1,000 simulated datasets, each based on samples of 50 individuals, for different true prevalence values. The boxplots represent the distribution of observed prevalence across simulations for each true prevalence. Variability is highest at intermediate prevalence levels and lowest at extreme values, consistent with theoretical expectations of binomial sampling variance.

Supplementary text 1: Considerations, recommendations and caveats when using SAMPLE:

SAMPLE is a practical tool designed to help users assess whether their field sampling efforts are sufficient to reliably estimate species prevalence. Several important factors should be considered when using SAMPLE, and they are listed below:

1. Species occurrence in nature is inherently variable across both time and space. SAMPLE does not account for this spatial or temporal heterogeneity, making it crucial for users to recognise and reflect this variability in their sampling strategy. If the collected data fails to capture this natural variation, the performance of SAMPLE will be compromised. Ultimately, the accuracy of SAMPLE's output is directly tied to the quality and representativeness of the data provided. Addressing these variations through thoughtful sampling design is essential for obtaining meaningful results.

2. The inherent stochastic nature of SAMPLE can introduce variability in both the estimated stability threshold (Fig. S2) and the prevalence rate (Fig. S3). To mitigate these fluctuations, it is advisable to conduct multiple replicates (we recommend a minimum of five runs) and assess convergence. Increasing the sampling effort is recommended until convergence is achieved. Some degree of variation will always persist, and it is important to keep in mind that the SAMPLE tool aims to provide a reliable proxy for the prevalence rate.

3. Certain species can be challenging to detect in nature. Examples include species employing deceptive camouflage strategies, cryptic species, very small organisms, those with hidden life cycles, and species living inside hosts. These organisms are often difficult to identify without invasive sampling and can be easily overlooked in field studies. Consequently, SAMPLE cannot address the inherent sampling challenges posed by the biology of such species.

4. Rare species present additional sampling challenges. Our simulations indicate that SAMPLE performs better at both low and high prevalence rates than at intermediate rates (Fig. S3), provided the samples collected are representative of

the actual prevalence. To further evaluate this trend, we conducted supplementary simulations mimicking the sampling process, focusing on how SAMPLE performed, particularly for low prevalence rates (Fig. S4). These simulations demonstrated that while error in estimated prevalence increases with sampling processes, the underlying trends remain consistent: lower errors are observed at both low and high prevalence levels compared to intermediate levels. This finding reflects the binomial-like nature of the sampling process. When prevalence is low, the variance in binomial trials is reduced, leading to less variability between successive estimates compared to intermediate prevalence rates. Consequently, SAMPLE can detect a stable prevalence more quickly at low prevalence levels. This behaviour aligns mathematically with the characteristics of binomial distribution variance and is thus expected. Reassuringly, these results suggest that if a species is rare, SAMPLE will likely indicate a low prevalence. When studying rare species using SAMPLE, it is advisable not to cease sampling until at least 5-6 hosts with symbionts or parasites have been collected.

In summary, SAMPLE is a valuable tool to add to the field ecologist's toolkit. It provides a user-friendly yet robust approach to guide sampling efforts and assess whether enough samples have been collected to estimate a stable prevalence rate. Users must always be mindful of the inherent variability in species occurrence, the stochastic nature of sampling, the challenges posed by difficult-to-detect species, and the complexities associated with rare species. To maximise the effectiveness of SAMPLE, we recommend adopting a comprehensive sampling strategy that includes multiple replicates and careful consideration of spatial and temporal variability. By integrating these practices, users can enhance the reliability of their prevalence estimates, ultimately contributing to a deeper understanding of species dynamics in their ecosystems.

Supplementary text 2: *SAMPLE relationship Between Prevalence and Sampling Stability*

In the main text, we discuss how the SAMPLE method determines the stability of prevalence estimates as the number of collected samples increases. A key observation from our simulations is that stability is reached more quickly for species with very low or very high prevalence values. Whilst this might seem counterintuitive at first, this outcome is expected given the mathematical properties of the underlying sampling process. Here, we provide a theoretical justification for this pattern, along with empirical validation using simulations.

Theoretical Basis: Prevalence and Variance in Sampling

When estimating the prevalence of a symbiont in a host population, each host sample represents a Bernoulli trial, where the probability of detecting the symbiont corresponds to the true prevalence, denoted as p. The cumulative outcome of multiple independent samples follows a hypergeometric distribution, which depends on three parameters:

- N: The total population size (number of host individuals).
- K: Number of hosts that are symbiont-positive.
- n: The number of host individuals sampled.

Since, in practice, the sample size n is much smaller than the total population size N, (i.e. $N >> n$), the hypergeometric distribution can be well approximated by the binomial distribution, with two parameters:

- *N:* The number of hosts sampled.
- *p:* The probability of encountering a symbiont (i.e., the true prevalence).

A crucial property of this function is that the variance is maximal when p = 0.5 and minimal when p is close to 0 or 1. This creates a reverse U-shaped relationship between prevalence and variance (Figure S5).

The variance properties of the binomial distribution have direct consequences for how quickly prevalence estimates stabilise as more samples are collected. This relationship explains why SAMPLE detects stable prevalence estimates more quickly for extreme prevalence values.

To illustrate this, we consider a scenario in which a researcher is sampling hosts to estimate the prevalence of a symbiont. Each collected host provides a new data point, and the researcher continues sampling until the estimated prevalence reaches a stable value.

When prevalence is very low, the variance in the number of symbiont-positive hosts remains small across different sampling attempts. If we imagine repeating the same sampling process multiple times under identical conditions, the number of symbiont-positive hosts observed in each attempt would show little fluctuation. The same holds for very high prevalence values, where most samples will contain the symbiont, again leading to little variation between repeated sampling attempts. This reduced variability makes it easier to detect a stable prevalence estimate after collecting only a modest number of samples.

By contrast, when prevalence is intermediate (e.g., around 50%), the number of symbiont-positive hosts fluctuates much more from one sampling attempt to the next. This is because variance in the binomial distribution is maximised at $p = 0.5$, meaning that each round of sampling is more likely to yield different results. As a consequence, more samples are required before a stable prevalence estimate emerges.

Illustration via simulations

To validate this theoretical expectation, we ran binomial simulations using known prevalence values (1%, 10%, 50%, 90%, and 99%). Each simulation was performed over 1,000 replicates using a fixed sample size of 50 host individuals per replicate. Figure S6 shows the distribution of observed prevalence across the 1,000 replicates. The results confirm the expected pattern: when prevalence is very low (1%) or very high (99%), the observed values are relatively consistent across replicates, with narrower interquartile ranges and fewer extreme values. In contrast, at intermediate prevalence levels,

particularly around 50%, there is much greater variability between replicates, leading to a wider spread in observed prevalence. This reflects the increased uncertainty in estimation when prevalence is moderate, compared to more extreme prevalence levels where the sample proportions are more stable.

Implication for SAMPLE

This trend is also evident in how quickly SAMPLE detects stable prevalence estimates. Because SAMPLE assesses stability by tracking changes in prevalence estimates as sample size increases, lower variance at extreme prevalence values enables stability to be reached more quickly. In contrast, at intermediate prevalence levels, larger fluctuations in estimates cause delays in stability detection. To demonstrate this effect, we conducted simulations using 1,000 replicates for known prevalence values ranging from 1% to 99%, sampling 50 individuals each time with SAMPLE's default parameters. The results, presented in Figure S2, show that SAMPLE consistently detects stability with fewer samples when prevalence is very low or very high, whereas detecting stability takes longer when prevalence is around 50%. These findings confirm that this pattern arises not from a methodological artifact but as a direct consequence of the variance properties of the binomial distribution.

Supplementary text 3: *The Influence of sampling on prevalence estimation in SAMPLE*

The sampling process can influence the accuracy of prevalence estimates. In many ecological systems, certain species may be difficult to sample or detect, leading to systematic underrepresentation in collected samples. SAMPLE relies on user-provided data, and its performance depends on the quality and representativeness of the sample. If the sample does not adequately capture the true prevalence—whether due to sampling bias, species biology, or detectability—this will naturally affect the accuracy of prevalence estimates.

To assess how sampling affects SAMPLE's output, we performed two additional sets of simulations. First, we simulated 100 individuals drawn from a binomial distribution, with the p parameter corresponding to prevalence levels ranging from 1% to 99%. This procedure was repeated 1,000 times for each prevalence rate. We then compared the true prevalence to the one estimated by SAMPLE, quantifying the error in the estimated prevalence. Since there was no additional sampling step, errors remained low, never exceeding 2.5% (Figure S3).

Next, to mimic the effect of sampling from a large population, we first generated a population of 100,000 individuals with fixed true prevalence values ranging from 1% to 99%. We then selected 100 random individuals without replacement from this population. This procedure was again repeated 1,000 times for each prevalence rate, and we computed the error in the estimated prevalence. The inclusion of this additional sampling step introduced greater variability in prevalence estimates, with errors reaching up to 12.5% (Figure S4).

Despite this increased variability, the overall trend remained consistent: estimation errors were smallest at both low and high prevalence levels and largest at intermediate ones. This pattern aligns with the binomial-like nature of the sampling process, as discussed in Text S2, and highlights how real-world sampling introduces additional noise into prevalence estimates. In essence, the second simulation highlights how real-world sampling processes can introduce more noise into prevalence estimates, reinforcing the importance of careful sampling design when using SAMPLE.
